# Efficacy and Safety of Azelaic Acid Nanocrystal-Loaded In Situ Hydrogel in the Treatment of Acne Vulgaris

**DOI:** 10.3390/pharmaceutics13040567

**Published:** 2021-04-16

**Authors:** Ivona Tomić, Sandra Miočić, Ivan Pepić, Dubravka Šimić, Jelena Filipović-Grčić

**Affiliations:** 1 Faculty of Pharmacy, University of Mostar, Matice hrvatske bb, 88000 Mostar, Bosnia and Herzegovina; ivona.tomic@farf.sum.ba (I.T.); dubravka.simic@mef.sum.ba (D.Š.); 2 R&D, PLIVA Croatia Ltd., TEVA Group Member, Prilaz baruna Filipovića 25, 10000 Zagreb, Croatia; Sandra.Miocic@pliva.com; 3 Faculty of Pharmacy and Biochemistry, University of Zagreb, Ante Kovačića 1, 10000 Zagreb, Croatia; ipepic@pharma.unizg.hr; 4 Department of Dermatology and Venereology, University Clinical Hospital of Mostar, Kralja Tvrtka bb, 88000 Mostar, Bosnia and Herzegovina

**Keywords:** azelaic acid, nanocrystals, hydrogels, acne, dermal application

## Abstract

Acne vulgaris is a common, multifactorial, inflammatory skin disease affecting the pilosebaceous unit. Topical therapy is the first choice in the treatment of mild to moderate acne, and azelaic acid (AZA) is one of the most commonly used drugs. The aim of this study was to evaluate the safety and efficacy of a low-dose azelaic acid nanocrystal (AZA-NC) hydrogel in the treatment of mild to moderate facial acne. The study was designed as a double-blind, randomized controlled trial. Patients were randomized to treatment with AZA-NC hydrogel, 10%, or AZA cream, 20%, administered in quantities of approximately 1 g twice daily for 8 weeks. Efficacy of therapy was measured by the number of lesions and safety by the frequency and severity of adverse events. At week 8, the success rate of treatment with AZA-NC hydrogel, 10%, was 36.51% (*p* < 0.001) versus 30.37% (*p* < 0.001) with AZA cream. At week 8, treatment with AZA-NC hydrogel, 10%, resulted in a significant reduction in total inflammatory lesions from baseline of 39.15% (*p* < 0.001) versus 33.76% (*p* < 0.001) with AZA cream, and a reduction in non-inflammatory lesions from baseline of 34.58% (*p* < 0.001) versus 27.96% (*p* < 0.001) with AZA cream, respectively. The adverse event rate was low and mostly mild.

## 1. Introduction

Acne vulgaris (AV) is a common, chronic, inflammatory disease of the pilosebaceous unit that usually manifests during puberty, but it can occur at any age, especially in women. It is associated with various clinical presentations such as comedones, papules, pustules and nodules [1,2].

The pathophysiology includes four primary factors that interact in the development of acne lesions: (i) androgen-dependent hyperseborrhea, (ii) abnormal follicular keratinization leading to comedones, (iii) follicular colonization by *Cutibacterium acnes* (*C. acnes*), and (iv) release of inflammatory mediators in the skin [3]. Acne lesions most commonly occur on the sites with the highest density of sebaceous glands, such as the face, neck, upper chest, shoulders, and back [4]. The inflammatory responses that stimulate and cause the different types of acne lesions include changes in the lipid profile of the sebaceous glands, dysseborrhea, stress, irritation, cosmetics, and possible dietary factors [5]. Due to the multifactorial pathophysiology, the treatment of AV should consider the full spectrum of pathogenic factors [6].

Acne can be treated topically, systemically, and by other treatments such as dermoabrasion, xenografting, autografting, and chemical peeling. Topical therapy is the first choice in acne treatment, especially for mild and moderate acne. Topical products are applied directly to the affected area, which has several advantages, such as reducing systemic absorption and increasing exposure of pilosebaceous units for treatment [7].

Most conventional topical formulations usually have a high incidence of side effects such as skin irritation, erythema, and pruritus, which reduce patient compliance and thus the effectiveness of therapy [8]. The incidence and intensity of skin irritation depends not only on the type of drug but also on its concentration, the vehicle formulation used and mode of administration [6]. Therefore, the ultimate goal in the field of topical acne treatment is to provide highly effective and well-tolerated therapy [6,9]. In addition, newly developed vehicle formulations should increase efficacy, e.g., by progressive follicular penetration of the active ingredient, and improve patient tolerability and compliance. A wide range of colloidal delivery systems are under development, including polymeric and solid lipid nanoparticles, liposomes, nanostructured lipid carriers and nanoemulsions. These can increase drug stability and facilitate the formulation of lipophilic drugs [6].

Azelaic acid (AZA), the compound of interest in this study, is a saturated dicarboxylic acid naturally produced by *Malassezia furfur*. It possesses antibacterial (*C. acnes*), anti-inflammatory and keratolytic activity. In addition, AZA competitively inhibits tyrosinase, DNA synthesis and mitochondrial enzymes. Therefore, it is an effective agent for the topical treatment of mild to moderate AV and postinflammatory hyperpigmentation [10]. The half-life of topical AZA is approximately 12 h, so patients should apply it to the affected areas twice daily. A favorable treatment outcome for acne vulgaris is usually achieved within 4 weeks [11]. AZA has a good safety profile and a low potential for irritation [6].

Dermal nanocrystals have emerged as a novel and versatile approach to overcome the skin barrier and enhance topical drug delivery. Previous research has shown that nanocrystals improve dermal bioavailability by increasing penetration through the skin. Due to the increased surface area of the nanocrystals, the dissolution rate increases, making it possible to maintain a large concentration gradient between the formulation and the skin, and thus forcing diffusion of the drug into the skin [12,13,14].

The small particle size of nanosuspensions is critical for their in vivo activity; however, due to their inherent instability, their formulation and preparation is challenging. Nanoscale suspensions are known to be thermodynamically unstable and, therefore, prone to agglomeration or recrystallization, in some cases even during processing or, more commonly, during shelf life. To prevent Ostwald ripening, a phenomenon in which large crystals grow at the expense of dissolving small crystals, the formulated active ingredients should be poorly soluble and the formulation should be stabilized with suitable stabilizers in sufficient concentrations [15,16].

Wet milling and high-pressure homogenization are the two most commonly used technologies for the preparation of nanosuspensions. Previous studies have shown the superiority of the wet milling process over high pressure homogenization in preventing crystal growth during processing and shelf life [16,17].

The aim of this study was to prepare an in-situ hydrogel of Pluronic^®^ F127 (P), hyaluronic acid (HA) and AZA in the form of nanocrystals (AZA-NC hydrogel). The particle size analysis of AZA nanocrystals suspension (AZA-NS) and the test of spreadability of AZA-NC hydrogel were also performed. In addition, the clinical efficacy of commercial AZA cream (Skinoren^®^) and AZA-NC hydrogel in reducing the number of inflammatory and non-inflammatory lesions in patients with mild to moderate AV was evaluated and compared.

## 2. Materials and Methods

### 2.1. Materials

Azelaic acid (AZA) and Pluronic^®^ F127 (P) were purchased from Sigma Aldrich (Chemie GmbH, Steinheim, Germany). Sodium hyaluronate (HA) and polysorbate 60 (P60) were purchased from Kemig (Zagreb, Croatia), commercial AZA 20% cream (Skinoren^®^) was purchased from Bayer (Zagreb, Croatia). Redistilled water was used for all experiments.

### 2.2. Preparation of the AZA Nanosuspensions (AZA-NS)

AZA was dispersed in an aqueous solution containing 0.3% (*w*/*w*) P60 (stabilizer) for 10 min at 600 rpm using a magnetic stirrer (IKA, Staufen, Germany) to form a coarse macrosuspension containing 5% (*w*/*w*) azelaic acid. The prepared suspension was further homogenized at 2000 rpm for 3 min using Silverson LM5 homogenizer (Silverson Machines Inc., East Longmeadow, MA, USA) to destroy any drug agglomerates present. To further reduce the particle size, the macrosuspension was processed on a Dyno-Mill Re-searchlab bead mill (Willy A. Bachofen AG, Muttenz, Switzerland) at 4000 rpm for 180 min. Yttrium Stabilized Zirconium Oxide Beads (Silibeads^®^ type ZY-P Pharma, Sigmund Linder GmBH, Warmensteinach, Germany) with a size of 0.1–0.2 mm and a loading of 55 mL per milling chamber were selected for the milling process. Milling was carried out in recirculation mode. Considering the temperature-dependent solubility of the azelaic acid, the temperature of the suspension was maintained at 25 ± 2 °C during milling. The nanosuspensions (AZA-NS) were stored at 5 °C until freeze drying.

### 2.3. Freeze Drying of the AZA Nanosuspensions

Nanosuspensions were solidified by freeze drying using an AdVantage Pro benchtop freeze dryer (SP Scientific, United States). The 4 mL nanosuspensions in glass vials were frozen at −55 °C for 180 min (freezing rate 1.5 °C/min), followed by a cooling step (at −30 °C for 180 min) and further lowering of the temperature to −55 °C for another 180 min (freezing rate 2.5 °C/min). Ice removal by primary drying of the nanosuspensions was performed by gradually increasing the shelf temperature in 10 °C increment from −55 °C to 0 °C (600 min holding time at −45 °C, −35 °C and −25 °C, 300 min holding time −15 °C, −5 °C and 0 °C) for 35 h in a vacuum of 150 µbar. Secondary drying was carried out for 120 min at temperatures of 10 and 20 °C, respectively, and a vacuum of 100 µbar to remove adsorbed water. The obtained lyophilizates were resuspended with redistilled water to form the nanosuspension (AZA-NS-FD) and analyzed for particle size.

### 2.4. Particle Size Analyses

Samples of AZA-NS were taken after 2 (AZA-NS-1), 3 (AZA-NS-2) and 4 h (AZA-NS-3) of grinding and analyzed for particle size distribution. Particle size distribution was also determined for a sample of the reconstituted AZA-NS-lyophilizate (AZA-NS-FD).

The particle size distribution of the nanosuspensions was determined by dynamic light scattering (DLS) using the Zetasizer NaNO ZS (Malvern Instruments, Malvern, UK). The results are presented as the mean intensity-weighted particle population diameter (z-average) and the polydispersity index (PDI), which is a measure of the width of the particle size distribution. Samples were diluted 50 times with water prior to measurement. Measurements were performed at a temperature of 25 °C and a measurement angle of 173°. The mean values were calculated from 3 individual measurements. The samples analyzed and the results obtained are shown in Table 1.

### 2.5. AZA-NC Hydrogel Formulation

The AZA-NC hydrogel was prepared using the FagronLab™ PRO unguator (Scheßlitz, Germany) according to our previous study [18]. Half of the required amount of redistilled water was placed in the original FagronLab™ vessel, followed by the addition of the 15% P, 1% HA and 10% lyophilizate of AZA nanocrystal suspension. Finally, the remaining amount of water was added. The mixing time was 26 min by 6 interchanging speed intervals (600 rpm vs. 1400 rpm) and times (485 s vs. 30 s vs. 510 s vs. 30 s vs. 485 s vs. 30 s).

### 2.6. Spreadability Test

The spreadability was investigated using a TTC spreadability device (HDP/SR*) at TA.XT*plus*C Texture Analyser (Stable Micro Systems Ltd., Godalming, United Kingdom) at 20 ± 5 °C. In this test, a 90° cone-shaped probe is pressed at a defined speed and depth into precisely matched cone filled with hydrogel or cream. The cone probe was lowered into the sample from a fixed position of 25 mm at a defined velocity of 3 mm/s and a defined stroke depth of 23 mm, so that the sample flows outward at 45° between the cones. The measurements were performed in duplicate. The textural properties of the formulation were calculated using the instrument software Texture Exponent. The resulting plot of force versus time provides data on the strength of the sample, measured as the maximum force reached in the curve, and the spreadability, which corresponds to the shear work, i.e., the area under the curve.

### 2.7. Patients and Clinical Study Design

The study included patients aged 14 to 40 years of both sexes diagnosed with mild to moderate AV. Exclusion criteria included some of the other forms of acne (e.g., severe, nodular, cystic), the presence of other skin conditions such as psoriasis and various forms of dermatitis, use of systemic or laser acne therapy in the previous month, known hypersensitivity to any ingredient in the test formulations, and pregnant and lactating women.

The study, in which 68 patients participated, was completed by 60 patients. They were randomly assigned to two groups and received either a commercial AZA cream (Skinoren^®^) containing 20% AZA or an AZA nanocrystal hydrogel containing 10% AZA (AZA-NC hydrogel). Both patients and their dermatologists were blinded to the type of treatment. Patients were instructed to apply the treatments to the affected areas twice daily (morning and evening) for eight weeks. They were also instructed not to use cleansers or cosmetics on the treated areas during the study period, and they provided written informed consent before participating in the study. The number of lesions (inflammatory, non-inflammatory, and total) was collected before treatment began, and the dermatologist determined the status of the condition at 4 and 8 weeks. No other acne medications were allowed during the study.

In this study, a standard lesion count was used to classify acne into four groups according to Hayashi et al. [19], corresponding to mild (0–5), moderate (6–20), severe (21–50) and very severe (>50).

Approval for the study was obtained from the relevant ethics committees. The study was conducted in accordance with the Declaration of Helsinki. Voluntary informed consent forms were signed by all patients and/or their parents or guardians.

### 2.8. Statistical Analysis

Data were stored in the database MS Excel 2000 and the statistical program SPSS (SPSS for Windows 17.0, SPSS, Chicago, IL, USA) was used for statistical analysis. The Student *t*-test and Chi-Square test were used to analyze the sociodemographic characteristics. Student *t*-tests were used to calculate the mean number of lesions at baseline and to compare study results between groups. The efficacy of treatment within a given group was analyzed using the paired-samples *t*-test. The sampling distribution was tested using the Kolmogorov–Smirnov test. The statistical significance level was set at 0.05.

## 3. Results and Discussion

### 3.1. AZA-NS Particle Size and PDI

To produce a sufficient amount of AZA nanocrystals for clinical supply, it was necessary to scale up the milling process, from the small low energy milling process used in our previous study [18] to the high energy agitator milling process.

Low-energy bead milling processes produce very fine particles with narrow size distributions, but the milling time is very long because of the relatively low energy input. In grinding processes based on high energy methods, such as in the agitator bead mill, the power density is much higher, resulting in a significant reduction in production time. This makes grinding in the agitator bead mill the process of choice for large-scale pharmaceutical applications [20]. Wet milling was chosen for the preparation of azelaic acid nanocrystals because previous studies have shown the superiority of the low energy bead milling process over high pressure homogenization in preventing crystal growth during processing and shelf life [16,17].

Compared to the previous study [18], the concentration of azelaic acid in the milling suspension was increased from 2% to 5% (*w*/*w*) to reduce the amount of dissolved drug fraction and increase stability, while increasing the efficiency of the milling process. Concentrations higher than 5% (*w*/*w*) resulted in a sharp increase in the viscosity of the suspension at the beginning of the milling, which made it impossible to continue the process.

Small beads of 0.1–0.2 mm and a high agitator speed were chosen to further improve the milling effect and reduce the processing time.

Stabilizers used in the formulation of nanosuspensions adsorb on the surfaces of nanocrystals and support the stability of the suspension by steric or electrostatic repulsion. It is reported in the literature that the optimum steric stabilizer to drug ratio should be in the range of 0.05:1 to 0.5:1 [21]. In the present study, the nonionic surfactant polysorbate 60 was selected for steric stabilization of the formulation at the ratio of surfactant to active ingredient of 0.06:1.

To determine the optimum milling time, samples were taken after 2, 3 and 4 h of continuous milling in recirculation mode and analyzed for particle size and PDI. The results are presented in Table 1.

The particle size distributions obtained were in agreement with the particle sizes reported for most nanosuspensions prepared by the wet milling process (ranging from 100 nm to 300 nm in size) [20,22]. No crystal growth was observed during the milling process. The process was successfully scaled using agitated ball mill technology, resulting in a 40-fold increase in processed suspension batch size (from 7.5 g to 300 g of suspension) and an 8-fold decrease in processing time (from 24 h to 3 h) compared to the previous study [18].

Since the particle size reached a plateau after 3 h of milling, the AZA-NS-2 nanosuspension was selected for further processing by freeze drying to prevent the occurrence of Ostwald ripening and crystal growth.

The freeze-drying step is a crucial process to ensure the stability of the nanocrystals. As previously reported, the nanocrystals tend to form non-dispersive agglomerates, especially during the freeze step, which affects the nanonization achieved during wet milling. To prevent this effect, the addition of a cryoprotective agent to the formulation may be necessary [23].

To enable homogenous incorporation of azelaic acid nanocrystals into the hydrogel, the aim of the freeze-drying process was to generate lyophilizates that can be readily reconstituted into nanocrystals close to their original size.

**Figure 1:**
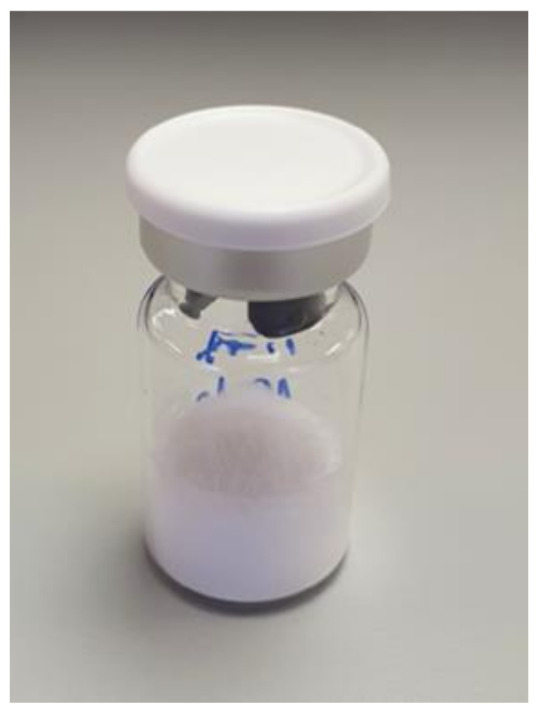
Azelaic acid nanocrystals lyophilizate.

The applied freeze-drying process resulted in a stable cake structure (Figure 1) that can be easily dispersed into nanocrystals without the need to add cryoprotectants to the formulation. The observed increase in crystal size from 144.8 nm to 269.9 nm after freeze drying could be attributed to the slight agglomeration of nanocrystals during the freeze-drying process. This change is acceptable because the resulting particle size was still in the target size range for which increased skin penetration was observed [12,24].

The satisfactory redispersibility properties of the cake can be attributed to the fast freezing rate and low concentration of nanocrystals in the suspension. As previously reported, during fast freezing, as the crystal growth of water progresses, the nanocrystals will not have sufficient time to move around and aggregate. Furthermore, a decrease in concentration leads directly to an increase in the distance between the particles, resulting in a reduced number of collisions between the particles and, thus, reducing aggregation [25].

Compared to a commercial 20% AZA cream that released 5% AZA in 480 min, an approximately 10-fold higher percentage of AZA was released from the AZA nanocrystal-loaded hydrogels in the same time period, as we have previously reported [18]. Therefore, we should expect the similar trend to be maintained here, as the particle size of the AZA nanocrystals, as well as the hydrogel formulation, remains similar.

### 3.2. Spreadability of AZA-NC Hydrogel

Spreadability is one of the essential properties of the topical formulation with respect to patient compliance. Even application of the product to the skin is easier and more acceptable to the patient if it has adequate spreadability. In addition, formulations with better spreadability can cover a larger area of the skin during application, which can improve the therapeutic effect [26].

Slight spreading is associated with low product strength. Texture profile analysis (TPA) showed that the strength and shear behaviour of AZA-NC hydrogel were 510.8 ± 2.0 g and 467.9 ± 28 g s, respectively, indicating that the strength of the hydrogel is low and it can spread at low shear [27]. Moreover, the strength and shear behaviour of AZA-cream were 3050.6 ± 61 g and 1079.0 ± 20.8 g s, respectively.

### 3.3. Efficacy of AZA-NC Hydrogel

The efficacy of AZA-NC hydrogel, 10%, compared with AZA cream, 20%, in the treatment of mild to moderate acne lesions on the face was evaluated in a double-blind, randomized clinical trial. The reason for the lower dosage of AZA than in all commercially available products (cream, 20%; gel, 15%; foam, 15%) was to reduce potential side effects such as burning, stinging, itching, dry skin, erythema, and irritation observed with 15% AZA gel and 20% AZA cream [11]. Furthermore, considering the in vitro results of AZA penetration into the skin from our previously published study [18], which showed that the AZA amount and total stratum corneum penetration depth were similar between 10% AZA gel and 20% AZA cream, a similar effect on acne lesions in vivo can be expected.

**Figure 2:**
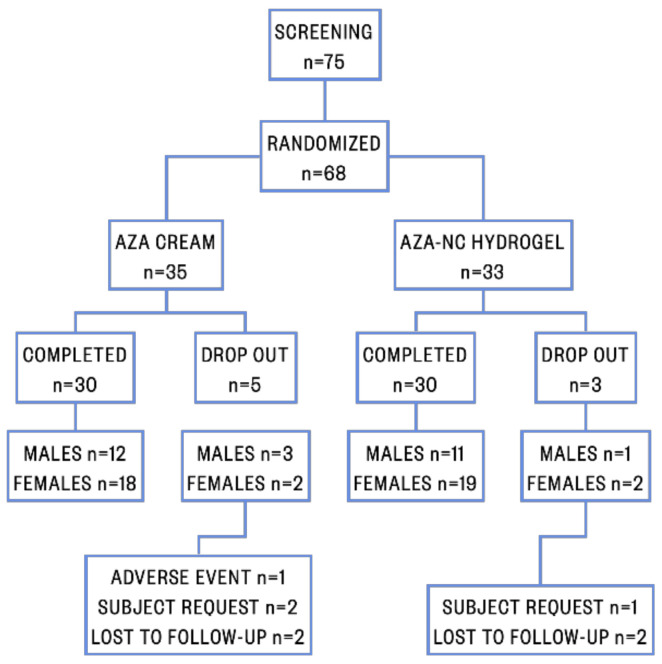
Patient flow diagram.

Seventy-five patients were initially screened, of whom 68 patients fulfilled the inclusion criteria and were randomized in two treatment groups: (i) thirty-five patients (51.47%) were randomized to AV treatment with AZA cream and (ii) thirty-three patients (48.53%) were randomized to AV treatment with AZA-NC hydrogel. Eight patients were excluded from the study; specifically, an adverse event occurred in one patient treated with AZA cream, three patients (two treated with AZA cream and one treated with AZA-NC hydrogel) requested to be excluded, and four patients (two treated with AZA cream and two treated with AZA-NC hydrogel) were lost to follow-up. The patient flow diagram is shown in Figure 2.

A total of 60 patients (23 males; 38.3% and 37 females; 61.7%) participated regularly in the study. The mean age of the patients was 18.83 ± 2.321 in the AZA cream treatment group and 18.57 ± 3.093 in the AZA NC hydrogel treatment group. The distribution by gender and all other sociodemographic characteristics, including work status and lifestyle habits such as smoking and alcohol consumption measured at baseline, were comparable and did not differ significantly between groups (Table 2).

The number of inflammatory, non-inflammatory, and total lesions was initially comparable, and the difference between treatment groups was not statistically significant (*p* > 0.05) (Table 3).

A comparison of the efficacy of reducing the number of inflammatory and non-inflammatory lesions after four- and eight-week treatment with AZA cream or AZA-NC hydrogel is shown in Table 4. The results show that both treatments were associated with a reduction in both inflammatory and non-inflammatory lesions. In addition, both treatments showed efficacy after four weeks of twice-daily AZA administration and a further increase in efficacy after eight weeks.

**Figure 3:**
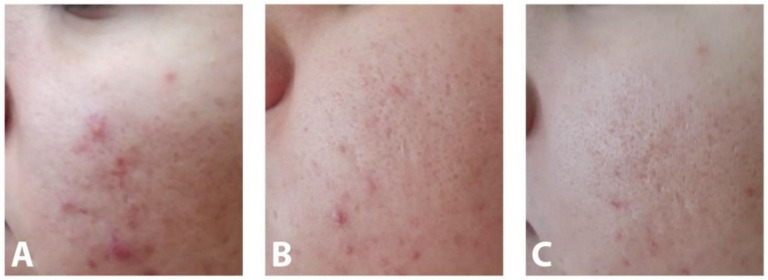
Patient with mild to moderate acne vulgaris at baseline (**A**), after four weeks (**B**) and after eight weeks (**C**) of treatment with AZA-NC hydrogel.

After four weeks of treatment, a reduction in both inflammatory and non-inflammatory lesions was observed in both groups of patients: in the AZA cream-treated group (11.66% reduction in inflammatory and 12.23% reduction in non-inflammatory lesions) and in the hydrogel group treated with AZA-NC (13.59% reduction in inflammatory and 13.75% reduction in non-inflammatory lesions). The same trend was observed after eight weeks of treatment. The reduction in the number of inflammatory and non-inflammatory lesions in the AZA cream-treated group was 33.76% and 27.96%, respectively, while in the AZA-NC-treated hydrogel group it was 39.15% and 34.58%, respectively. Figure 3 illustrates the reduction in lesion numbers during the treatment period with AZA-NC hydrogel.

The results are in agreement with those of Schaller et al. [28], who studied the effect of 20% azelaic acid cream (Skinoren^®^ cream) on mild to moderate AV and showed that application twice daily for 8 weeks resulted in a 39.8% reduction in the total number of lesions, while application for 12 weeks resulted in a 53.9% reduction.

The results of the drug on the reduction in the number of inflammatory, non-inflammatory, and total lesions within each study group after eight weeks of treatment are shown in Table 5. After eight weeks of AZA administration twice daily, a statistically significant (*p* < 0.001) reduction in inflammatory, non-inflammatory and, thus, total lesions, was achieved in both study groups. Moreover, high values of partial η^2^ showed a significant effect of both treatments on the reduction in the number of lesions. In the group of patients applying AZA cream, the values of partial η^2^ for inflammatory, non-inflammatory and total lesions were 0.710, 0.745 and 0.797, respectively, while in the group of patients applying AZA-NC hydrogel, the partial η^2^ values were higher and were 0.794, 0.848 and 0.846 for inflammatory, non-inflammatory and total lesions, respectively.

In conclusion, both AZA cream and AZA-NC hydrogel treatments showed progressive and continuous improvement in mild to moderate facial skin AV throughout the study period. Moreover, both treatments had a better effect on inflammatory compared to non-inflammatory lesions, which is consistent with the results reported by Schaller et al. [28] and the anti-inflammatory effect of AZA [29]. Compared to AZA cream, AZA-NC hydrogel treatment showed the same effect, although the AZA concentration in the cream was twice as high.

It has been shown that the efficacy of topically applied AZA in the treatment of acne lesions can be maintained in its reduced concentrations when it has been nanonized. It may also be possible to further reduce AZA concentrations and its side effects by partially replacing it with some substances of natural origin. Considering the antimicrobial and anti-inflammatory properties of essential oils, a recent study indicates their use and efficacy in topical acne treatment [30] in combination with tretinoin in a cream formulation. In addition, compared to synthetic agents, essential oils are safer and less toxic penetration enhancers for both hydrophilic and lipophilic agents [31], which can potentially reduce the possibility of side effects.

## 4. Conclusions

The AZA nanocrystal suspensions were successfully obtained by the agitated ball mill technology, from which the easily redispersible AZA nanocrystals were obtained by freeze drying without the need of adding cryoprotectants, which allowed their homogeneous incorporation into the hybrid poloxamer/hyaluronic acid in situ hydrogel. The 10% AZA-NC hydrogel exhibited good spreadability properties, allowing uniform application to the skin and good patient compliance.

The results of a randomized, double-blind clinical trial showed that the 10% AZA-NC hydrogel was as effective as the 20% AZA cream in treating mild to moderate facial skin AV. It effectively reduced the number of inflammatory and non-inflammatory lesions and showed an initial clinical effect after only four weeks of continuous use, although the benefits become more apparent after longer continuous treatment. Hydrogel AZA-NC, 10%, appears to show a favorable safety profile and improved efficacy in the treatment of mild to moderate AV.

## Figures and Tables

**Table 1:** The z-average particle size and polydispersity index (PDI) of azelaic acid nanosuspensions (AZA-NS).

AZA-NS	Grinding Time (h)	z-Average (nm ± SD)	PDI
AZA-NS-1	2	188.7 ± 34.1	0.49
AZA-NS-2	3	148.1 ± 6.5	0.40
AZA-NS-3	4	144.8 ± 4.7	0.36
AZA-NS-FD *	3	269.9 ± 14.8	0.43

* sample of the reconstituted AZA-NS-lyophilizate.

**Table 2:** Sociodemographic patient characteristics.

Patient Characteristic	AZA Cream	AZA-NC Hydrogel	*p*-Value
Age (mean ± SD)	18.83 ± 2.321	18.57 ± 3.093	0.707
Gender (n; %)			<1
M	12; 40.0%	11; 36.7%	
F	18; 60.0%	19; 63.3%	
Smoking (n; %)	12; 40.0%	11; 36.7%	<1
Alcohol consumption (n; %)	9; 30.0%	9; 30.0%	<1
Work status (n; %)			
Employed	2; 6.7%	4; 13.3%	
Student	20; 66.7%	13; 43.3%	
Pupil	8; 26.7%	13; 43.3%	

**Table 3:** The mean number of lesions at baseline in all patients.

Lesions type_week	AZA Cream Mean ± SD	AZA-NC Hydrogel Mean ± SD	*p*-Value
Inflammatory lesions_0	5.43 ± 3.421	6.13 ± 3.501	0.437
Non-inflammatory lesions_0	7.63 ± 4.189	8.00 ± 3.877	0.726
Total lesions_0	13.07 ± 7.080	14.13 ± 6.872	0.556

**Table 4:** The mean number and percentage reduction in inflammatory and non-inflammatory lesions after 4 and 8 weeks of treatment.

Lesions type_week(s)	AZA Cream Mean ± SD	AZA-NC Hydrogel Mean ± SD	*p*-Value
Inflammatory lesions_0	5.43 ± 3.421	6.13 ± 3.501	0.437
Inflammatory lesions_4	4.80 ± 3.253	5.30 ± 3.153	0.548
Inflammatory lesions_8	3.60 ± 2.430	3.73 ± 2.612	0.839
Diff. inflammatory lesion 0–4	0.63 ± 0.556	0.83 ± 0.791	0.262
Reduction_4 (%)	11.66	13.59	
Diff. inflammatory lesion 0–8	1.83 ± 1.206	2.40 ± 1.248	0.079
Reduction_8 (%)	33.76	39.15	
Non-inflammatory lesions_0	7.63 ± 4.189	8.00 ± 3.877	0.726
Non-inflammatory lesions_4	6.73 ± 3.823	6.90 ± 3.614	0.863
Non-inflammatory lesions_8	5.50 ± 3.330	5.23 ± 3.137	0.751
Diff. non-inflammatory lesion 0–4	0.93 ± 0.740	1.10 ± 0.845	0.420
Reduction_4 (%)	12.23	13.75	
Diff. non-inflammatory lesion 0–8	2.13 ± 1.279	2.77 ± 1.194	0.052
Reduction_8 (%)	27.96	34.58	

**Table 5:** Efficacy of treatment on reducing the number of inflammatory, non-inflammatory, and total lesions within a given group after eight weeks.

Lesions type_week(s)	AZA Cream Mean ± SD	*p*-Value	η^2^	AZA-NC Hydrogel Mean ± SD	*p*-Value	η^2^
Inflammatory lesions_0	5.43 ± 3.421	<0.001	0.710	6.13 ± 3.501	<0.001	0.794
Inflammatory lesions_8	3.60 ± 2.430			3.73 ± 2.612		
Reduction in lesion count 0–8 (%)	33.76			39.15		
Non-inflammatory lesions_0	7.63 ± 4.189	<0.001	0.745	8.00 ± 3.877	<0.001	0.848
Non-inflammatory lesions_8	5.50 ± 3.330			5.23 ± 3.137		
Reduction in lesion count 0–8 (%)	27.96			34.58		
Total lesions_0	13.07 ± 7.080	<0.001	0.797	14.13 ± 6.872	<0.001	0.846
Total lesions_8	9.10 ± 5.422			8.97 ± 5.189		
Reduction in lesion count 0–8 (%)	30.37			36.51		

## Data Availability

Data are included in the article.
